# Supplementary material for: 5′ Region Large Genomic Rearrangements in the BRCA1 Gene in French Families: Identification of a Tandem Triplication and Nine Distinct Deletions with Five Recurrent Breakpoints
Source: Cancers (Basel). 2021 Jun 25;13(13):3171. doi: 10.3390/cancers13133171 (PMC8268747; doi:10.3390/cancers13133171)
Supplement: Supplementary file 1 [file cancers-13-03171-s001.zip › Supplementary_figures-final_cancers.pptx]

## Slide 1
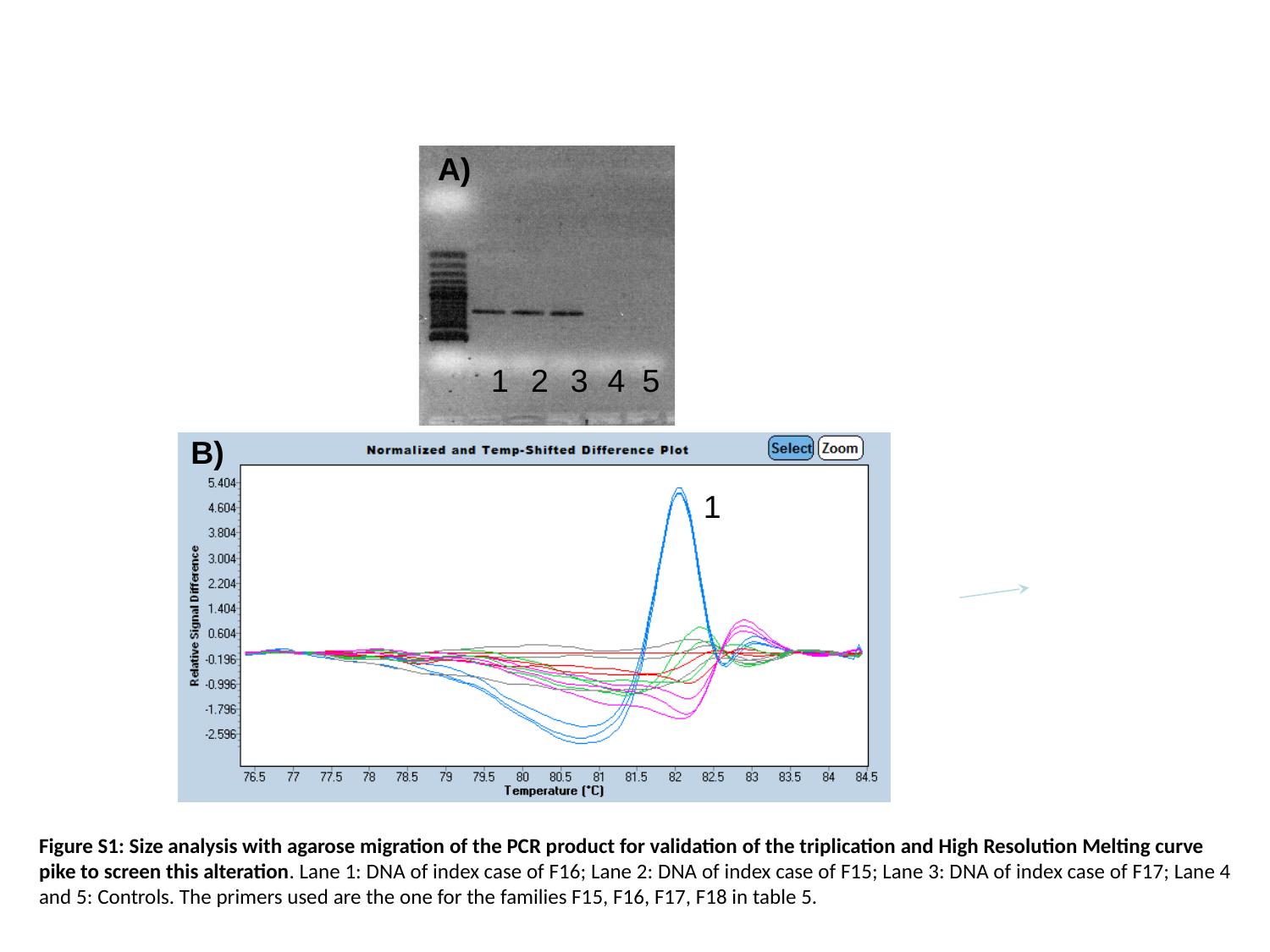

A)
1
2
3
4
5
B)
1
Figure S1: Size analysis with agarose migration of the PCR product for validation of the triplication and High Resolution Melting curve pike to screen this alteration. Lane 1: DNA of index case of F16; Lane 2: DNA of index case of F15; Lane 3: DNA of index case of F17; Lane 4 and 5: Controls. The primers used are the one for the families F15, F16, F17, F18 in table 5.

## Slide 2
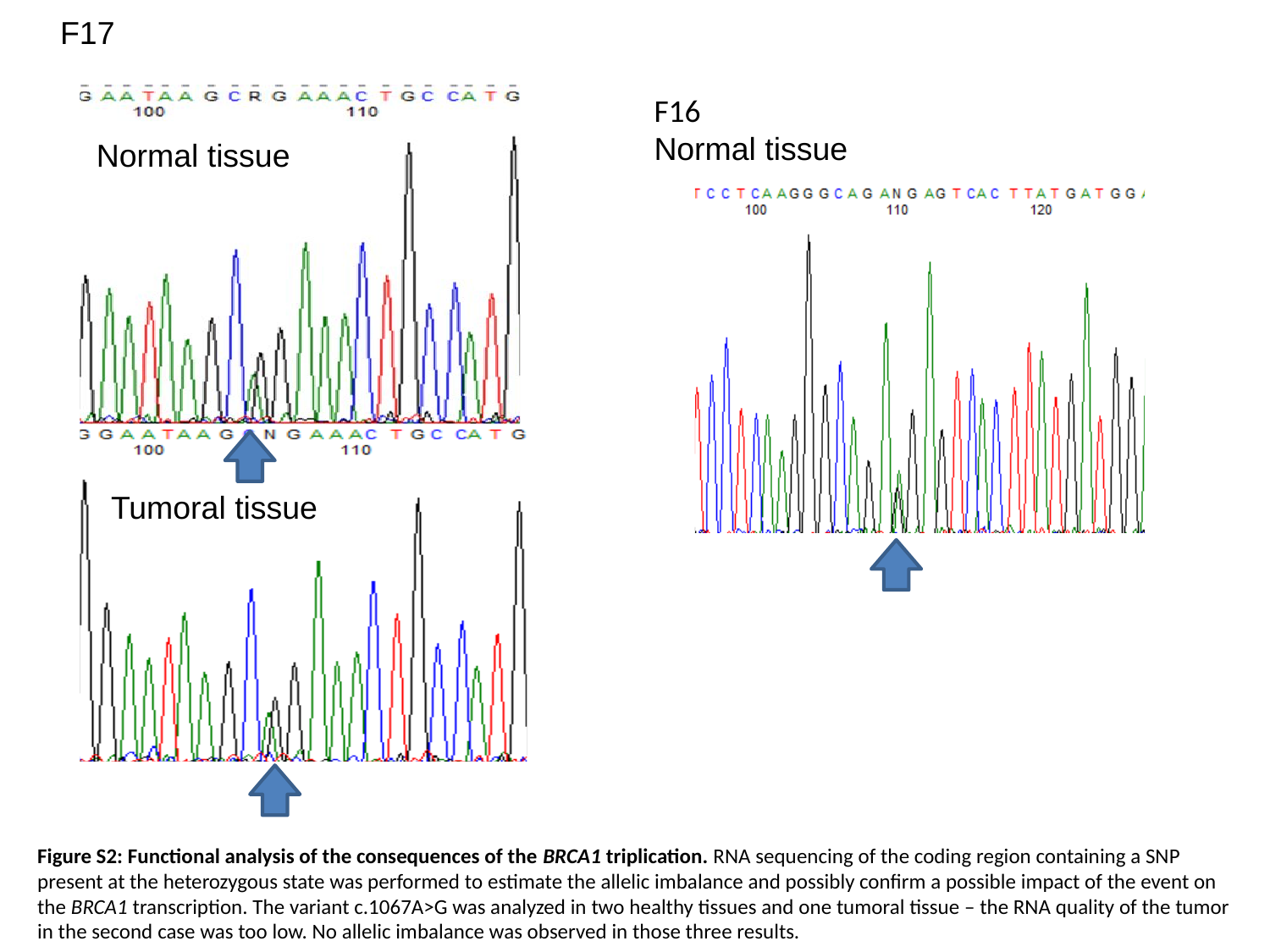

F17
F16
Normal tissue
Normal tissue
Tumoral tissue
Figure S2: Functional analysis of the consequences of the BRCA1 triplication. RNA sequencing of the coding region containing a SNP present at the heterozygous state was performed to estimate the allelic imbalance and possibly confirm a possible impact of the event on the BRCA1 transcription. The variant c.1067A>G was analyzed in two healthy tissues and one tumoral tissue – the RNA quality of the tumor in the second case was too low. No allelic imbalance was observed in those three results.

## Slide 3
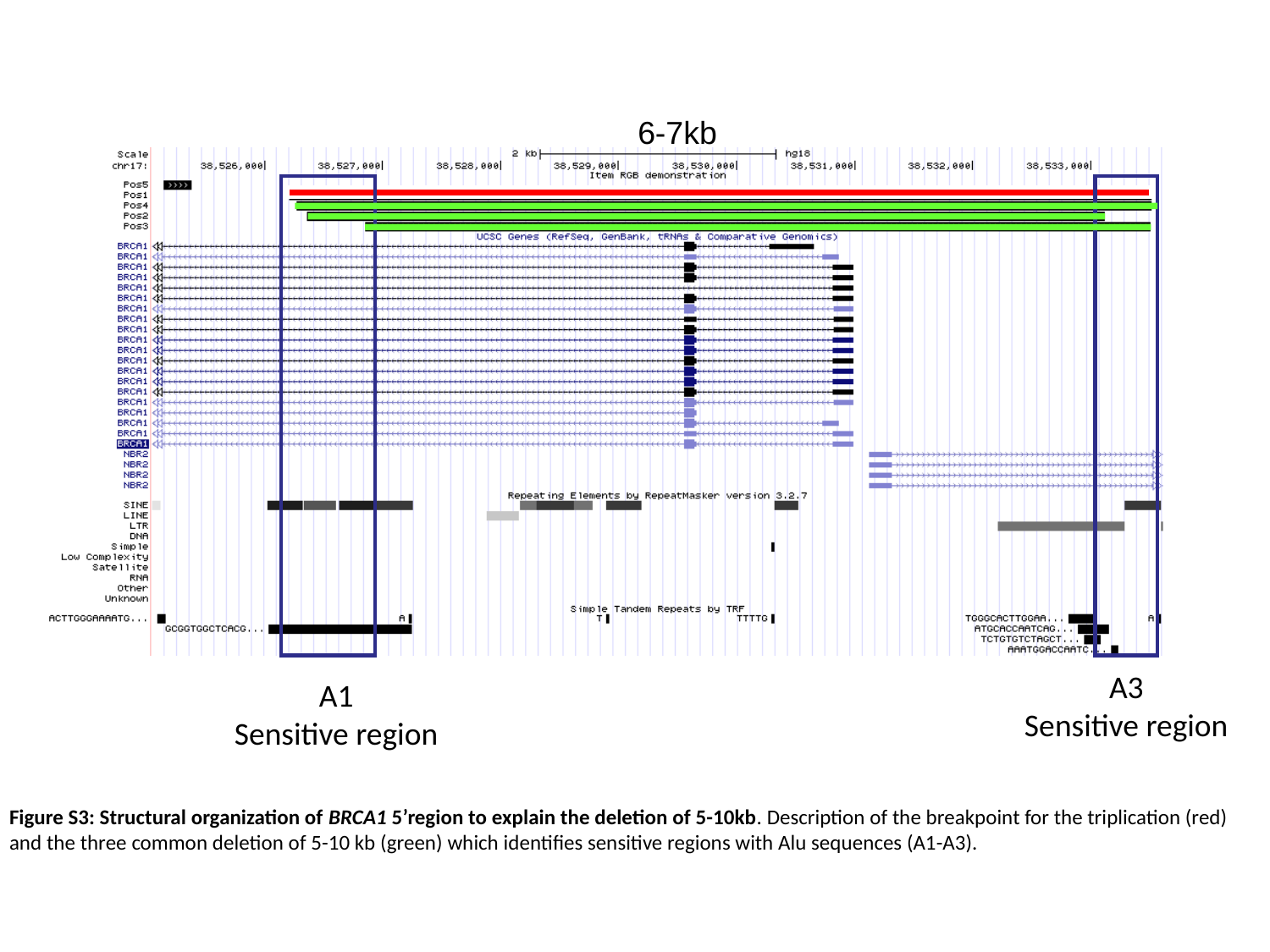

6-7kb
A3
Sensitive region
A1
Sensitive region
Figure S3: Structural organization of BRCA1 5’region to explain the deletion of 5-10kb. Description of the breakpoint for the triplication (red) and the three common deletion of 5-10 kb (green) which identifies sensitive regions with Alu sequences (A1-A3).
