# Supplementary material for: 5′ Region Large Genomic Rearrangements in the BRCA1 Gene in French Families: Identification of a Tandem Triplication and Nine Distinct Deletions with Five Recurrent Breakpoints
Source: Cancers (Basel). 2021 Jun 25;13(13):3171. doi: 10.3390/cancers13133171 (PMC8268747; doi:10.3390/cancers13133171)
Supplement: Supplementary file 1 [file cancers-13-03171-s001.zip › Table S2.pdf]

Table S2: French COVAR group collaborators (COsegregation of VARiants in the *BRCA1/2* and *PALB2* genes)

(https://clinicaltrials.gov/ct2/show/NCT01689584)

Town (France)	Center	Members
Amiens	CHU Amiens	Florence Amram, Michèle Mathieu-Dramard, Gilles Morin, Bénédicte Demeer
Angers	ICO - Centre Paul Papin Service de Génétique Médicale	Claire Le Hetet, Olivier Ingster, Marie-Emmanuelle Morin-Meschin
Angoulême	Centre Hospitalier d'Angoulême Oncogénétique	Paul Gesta, Stéphanie Chieze-Valero
Avignon	Institut Sainte-Catherine Oncogénétique	Julie Mestre, Hélène Dreyfus
Besançon	CHU Besançon ; Hôpital Saint-Jacques ; Service de Génétique Biologique	Céline Populaire, Marie-Agnès Collonge-Rame, Jean-Luc Bresson,
Bordeaux	Institut Bergonié	Michel Longy; Emmanuelle Barouk-Simonet, Pierre Macquere, Sabine Raad, Virginie Bubien; Anais Dupre, Henri Margot, Françoise Bonnet, Virginie Bubien, Natalie Jones, Nicolas Sévenet
Bordeaux	CHU- Groupe Hospitalier Pellegrin	Cécile Gaudebout, Noémie Bronnec, Julie Tinat
Bourges	CH Jacques Coeur	Isabelle Mortemousque
Brest	CHU de Brest	Julie Ménard, Séverine Audebert-Bellanger
Caen	Centre François Baclesse	Pascaline Berthet; Céline Heude, Agathe Ricou, Sophie Krieger, Laurent Castéra, Dominique Vaur
Chambery	Centre Hospitalier Hôtel Dieu Laboratoire de Génétique	Sandra Ferrer, Charlotte Leblanc
Clermont-Ferrand	Centre Jean Perrin - Département d'Oncogénétique	Nancy Uhrhammer, Maud Privat, Mathilde Gay-Bellile, Yves-Jean Bignon,
Dijon	CHU de Dijon Hôpital d'Enfants Service de Génétique Médicale	Vincent Goussot, Sophie Nambot, Salima El Chehadeh; Caroline Jacquot-Sawka, Laurence Faivre-Olivier
Fort de France, Martinique	CHU de Fort de France Unité d'Oncogénétique	Odile Béra, Sarah Malsa
Grenoble	CHU de Grenoble	Helene Dreyfus, Clémentine Legrand, Gabrielle Le Guyadec, Anne Béchet, Marie Bidart, Dominique Leroux
La Réunion	CHU Sud Réunion	Hanitra Ranjatoelina
La Rochelle	Centre Hospitalier Saint-Louis Oncogénétique	Paul Gesta, Hakima Lallaoui
Le Havre	Hôpital Flaubert Unité de Génétique	Valérie LAYET
Lille	Centre Oscar Lambret, Departement de cancérologie Générale	Audrey Mailliez, Berengere Legendre, Anne-Laure Conoy, Joelle Fournier, Françoise Revillion
Lille	CHRU LILLE Hôpital Jeanne de Flandre Service de Génétique Clinique	Sophie Lejeune, afane Brahimi, Laurence Bellengier, Sylvie Manouvrier-Hanu
Limoges	CHU Dupuytren Oncogénétique	Laurence Vénat-Bouvet, Anais Grangier, Sandrine Lavau-Denes

Lyon	Centre Léon Bérard Lyon	Valérie Bonadona; Sylvie Mazoyer ; Olga Sininilkova, Mélanie Léone, Adrien Buisson, Sandrine Handallou, Nadia Boutry-Kryza, Christine Lasset
Lyon	Hospices civils de Lyon	Juliette Santenard, Sophie Giraud
Marseille	Institut Paoli-Calmettes Département d'Oncologie Génétique	Audrey Remenieras, Doriane Livon, Hagay Sobol, Tetsuro Noguchi, Cornel Popovici, Violaine Bourdon, François Eisinger, Jessica Moretta-Serra, Catherine Noguès
Marseille	CHU La Timone, Département de Génétique	Hélène Zattara
Montpellier	CHU Arnaud de Villeneuve - Service de Génétique	Jean-Marc Rey, Laurence Lode, Pierre-Olivier Harmand, Pascal Pujol, Paul Vilquin, Virginie Galibert, Isabelle Coupier
Nancy	Centre Alexis Vautrin; Oncogénétique	Elisabeth Luporsi, Dominique Saint-Dizier, Charlotte Benigni
Nancy	CHU Nancy ; Hôpital Brabois ; Service de Génétique	Myriam Bronner, Joanna Sokolowska-Gillois, Christophe Philippe, Sarab Lizard, Philippe Jonveaux
Nantes	ICO - Centre René Gauducheau Unité d'Oncogénétique	Capucine Delnatte, Virginie Guibert, Céline Garrec, Fabrice Airaud, Stéphane Bézieau, Estelle Cauchin, Caroline Abadie
Nantes	Centre Catherine de Sienne Oncogénétique	Alain Lortholary, Alica Yvard
Nice	Centre Antoine Lacassagne - Nice	Véronique Mari; Jennifer Oudard, Marc Frenay
Nimes	CHRU Caremeau, Oncogénétique	Jean Chiesa, Audrey Combes
Niort	Centre Hospitalier de Niort Oncogénétique	Jeanne Triquard, Stéphanie Chiez-Valéro, Paul Gesta
Orléans	Hôpital de la Source Génétique	Isabelle Mortemousque, Julie Genot, Edouard Cottereau
Paris	Institut Curie site Paris Génétique Oncologique	Virginie Caux-Moncoutier, Lisa Golmard, Claude Houdayer, Muriel Belotti, Antoine Depauw, Jessica Le Gall, Maud Blanluet, Mathias Schwartz, Agnès Collet, Alice Fiévet, Caroline Lecerf, Marine Lementec, Anais Dupre, Mathilde Warcoin, Chrystelle Colas, Bruno Buecher, Marion Gauthier-Villars, Dominique Stoppa-Lyonnet
Paris	Groupe Hospitalier Pitié-Salpêtrière - Laboratoire d'Oncogénétique et d'Angiogénétique	Florence Coulet, Mathilde Warcoin, Erell Guillerm, Noemie Basset, Florent Soubrier, Veronica CUSIN, Patrick BENUSIGLIO
Paris	Hôpital Tenon Oncogénétique Clinique	Anne Fajac, Florent Soubrier, Patrick BENUSIGLIO, Nathalie Chabbert Buffet
Paris	HEGP	Geraldine Perkins, Diane Molière, Anne-Marie Birot, Pierre-Laurent Puig
Paris	Hôpital Saint-Antoine, Département de génétique	Jeanne NETTER-COTI, Patrick BENUSIGLIO
Paris	Hôpital Saint-Louis – Oncogénétique	Odile Cohen-Haguenauer, Paul Vilquin, Daniel Toledano
Pointe à Pitre, Guadeloupe	CHU de Pointe à Pitre	Marilyn Lackmy-Port-Lis
Poitiers	CHU La Milétrie Oncogénétique	Paul Gesta, Stéphanie Chieze-Valero, Brigitte Gilbert-Dussardier
Reims	Polyclinique de Courlancy	Nathalie Heil, Frédérique Carré-Pigeon
Reims	Institut Jean Godinot ; Service de	Tan Dat Nguyen; Helene Larbre, Fanny

	Génétique et Biologie de la Reproduction	Brayotel, David Bonnerave; Aude-Marie Savoye, Philippe Guilbert
Reims	CHU de Reims ; Service de Génétique	Dominique Gaillard ; Céline Poirsier ; Mozelle-Nivoix Monique, Anne Durlach
Rennes	Centre Eugène Marquis	Louise Crivelli, Vanessa Colombert, Enora Lejeune, Caroline Abadie
Rouen	CHU de Rouen<br>Unité de Génétique Clinique	Annick Rossi, Isabelle Thevenet, Thierry Frebourg
Saint-Etienne	CHU Saint-Etienne Hôpital Nord	Marine Lebrun, Fabienne Prieur
Saint-Cloud	Institut Curie<br>site Hôpital René Huguenin<br>Epidémiologie Clinique	Emmanuelle Fourme, Claire Saule, Ophélie Bertrand, Helene Delhommel, Chrystelle Colas
Strasbourg	Centre Paul Strauss ; Laboratoire de Biologie Tumorale	Laurène Rebattu, Danièle Muller, Hélène Nehme-Schuster
Strasbourg	Hopital Universitaire de Strasbourg	Chistine Maugard
Toulouse	Institut Claudius Regaud<br>Laboratoire d'Oncogénétique	Viviane Feillel; Norbert Lignon, Christine Toulas, Laurence Gladieff
Tours	CHU Bretonneau ; Service de Génétique ; Génétique	Isabelle Mortemousque
Troyes	CHG Troyes	Monique Mozelle
Valence	Centre Hospitalier de Valence	Marie-Noelle Bonnet-Dupeyron
Villejuif	Institut Gustave Roussy	Marine Guillaud-Bataille, Marina Dimaria, Sophie Villebasse, Odile Cabaret, Alice Fievet, Etienne Rouleau, Brigitte Bressac-De Paillerets, Olivier Caron
